# Extensive colonic necrosis following cardiac arrest and successful cardiopulmonary resuscitation: report of a case and literature review

**DOI:** 10.1186/1749-7922-7-35

**Published:** 2012-11-16

**Authors:** Iraklis E Katsoulis, Alexia Balanika, Maria Sakalidou, Ioanna Gogoulou, Athanasios Stathoulopoulos, Michael K Digalakis

**Affiliations:** 1 1st Surgical Department, Asklepieio General Hospital, Voula, Greece; 2 Computed Tomography Department, Asklepieio General Hospital, Voula, Greece

**Keywords:** *Colonic necrosis*, *Non-occlusive ischaemia*, *CPR*

## Abstract

Non-occlusive colonic ischaemia is a recognized albeit rare entity related to low blood flow within the visceral circulation and in most reported cases the right colon was affected. This is the second case report in the literature of extensive colonic necrosis following cardiac arrest and cardiopulmonary resuscitation (CPR). A 83-year-old Caucasian woman was admitted to our hospital due to a low energy hip fracture. On her way to the radiology department she sustained a cardiac arrest. CPR started immediately and was successful. A few hours later, the patient developed increasing abdominal distension and severe metabolic acidocis. An abdominal multidetector computed tomography (MDCT) scan was suggestive of intestinal ischaemia. At laparotomy, the terminal ileum was ischaemic and extensive colonic necrosis was found, sparing only the proximal third of the transverse colon. The rectum was also spared. The terminal ileum and the entire colon were resected and an end ileostomy was fashioned. Although the patient exhibited a transient improvement during the immediate postoperative period, she eventually died 24h later from multiple organ failure. Histology showed transmural colonic necrosis with no evidence of a thromboembolic process or vasculitis. Therefore, this entity was attributed to a low flow state within the intestinal circulation secondary to the cardiac arrest.

## Introduction

Non-occlusive colonic ischaemia is a recognized albeit rare entity related to low blood flow within the visceral circulation**.** Post-traumatic shock-associated colonic ischaemia has been previously reported in young, healthy patients and has involved primarily the right colon in most instances [1-5]. Only a few cases of extensive non-occlusive colonic gangrene have been reported [6-10]. This is the second case report of colonic necrosis secondary to cardiac arrest followed by successful CPR.

### Case presentation

A 83-year-old Caucasian woman was admitted to our hospital due to a low energy fracture of her left hip. The initial assessment in the Emergency Department revealed pallor, tachycardia and a systolic blood pressure of 110 mmHg. Her past medical history included coronary artery disease, arterial hypertension and depression for which the patient was under medication over the last three years. On her way to the radiology department the patient sustained a cardiac arrest. Cardiopulmonary resuscitation (CPR) started immediately and she was intubated. CPR was successful and the patient was subsequently transferred to the Intensive Care Unit (ICU). During her stay in the ICU, the vasoconstricting agent noradrenaline had to be installed in order to support her circulation and after a few hours she developed increasing abdominal distension and severe metabolic acidocis (PH = 7.14 with a Standard Base Excess = − 13.6 mEq/L). The patient underwent a multidetector computed tomography (MDCT) examination from the dome of the diaphragm to the symphysis pubis with a 6-row multidetector CT (Philips, Brilliance 6); using biphasic CT protocol for the abdomen without oral contrast administration. A 120 ml non-ionic contrast medium (350mg/ml iobitridol) and 50 ml of normal saline flush were administered intravenously with a power injector at a flow rate 3mls/s, with scan delay for starting arterial and portal-venous phases at 10s and 100s, respectively. Image acquisitions parameters were: 5 mm slice thickness, slice collimation of 1.5 mm, pitch 1, 140 kV and 120mAs.

**Figure 1:**
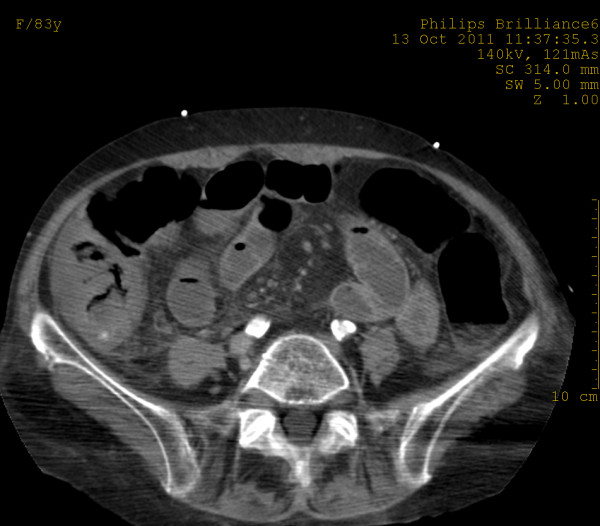
**Axial CT image at arterial phase demonstrates a thickened caecal wall.** A focal area of high attenuation suggesting active bleeding is seen in the lumen of the caecum.

**Figure 2:**
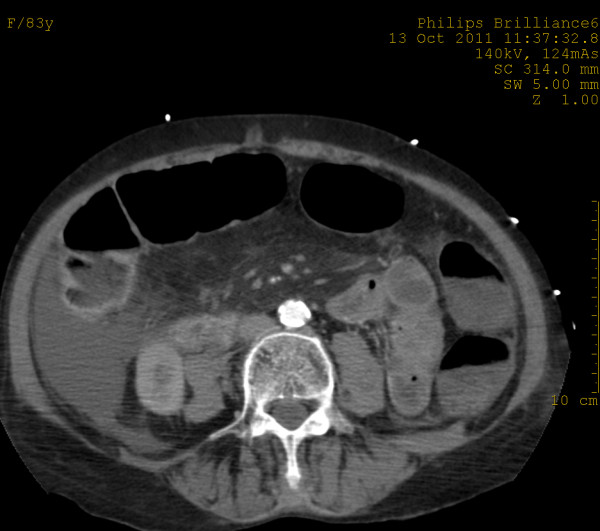
Axial CT image at venous phase shows intraperitoneal fluid and pericaecal fat stranding.

In the arterial phase, MDCT showed at least two focal areas of high attenuation (> 90 HU) within the lumen of the ascending colon and caecum suggestive of active bleeding [11]. Axial CT images at the level of the upper and the middle abdomen demonstrated thickened caecal and ascending colon wall (up to 11.5 mm) [12,13] with increased density due to intravenous contrast enhancement, pericaecal fat stranding and low-attenuation areas of intraperitoneal fluid at the root of the mesentery, at the perihepatic and Morrison’s spaces (Figures 1,2). No endoluminal defect of mesenteric arteries and veins was noted.

The above CT findings were suggestive of intestinal ischaemia and in association with the patient’s deterioration an exploratory laparotomy was undertaken which revealed ischaemia of the terminal ileum and extensive colonic necrosis sparing only the proximal third of the transverse colon. The rectum was also spared. The terminal ileum and the entire colon were resected and an end ileostomy was fashioned through the right abdominal rectus muscle sheath. The rectal stump was closed and left in the pelvis (Hartmann’s pouch). Although the patient exhibited a transient improvement during the immediate postoperative period, she eventually died 24h later from multiple organ failure. Histology showed transmural colonic necrosis without evidence of a thromboembolic process or vasculitis. Therefore, the aetiology was felt to be a low flow state within the intestinal circulation most likely secondary to the cardiac arrest.

## Discussion

The colon presents weak points on blood supply and poor autoregulation of blood flow that constitute the main predisposing factors for splachnic vasoconstriction and non-occlusive ischaemia [1]. Following experiments on flow characteristics within the mesenteric circulation when subjected to changing haemodynamics, Nikas D *et al.* found that the colon has the greatest sensitivity to hypotension [14].

An experimental model has also been used involving cardiogenic shock produced by pericardial tamponade [15]. This was associated with marked reductions in the intestinal blood flow. More recently Toung *et al.*[16], in another experimental model, involved variable degrees of hypovolaemic shock produced by graded levels of haemorrhage, from 12.5 to 50% of the calculated blood volume. This was associated with disproportional mesenteric ischaemia due to mesenteric vasoconstriction. They concluded that like cardiogenic shock, haemorrhagic shock generates selective mesenteric ischaemia by producing a disproportionate mesenteric vasospasm that, which is mediated primarily by the renin-angiotensin axis.

Both haemorrhagic and cardiogenic shocks can result in decreased perfusion pressure, prompting selective vasoconstriction of the mesenteric arterioles to maintain perfusion pressure of the vital organs, at the selective expense of the mesenteric organs. The response to any of these conditions can, variably and unpredictably, cause haemorrhagic gastric stress erosions, non-occlusive mesenteric ischaemia of the small bowel, ischaemic colitis, ischaemic hepatitis, acalculous cholecystitis, and ischaemic pancreatitis. Injury to the mesenteric organs can also initiate the systemic inflammatory response syndrome and, consequently, multiple organ failure [17,18].

Post-traumatic shock-associated colonic ischaemia has been previously reported in young, healthy patients and has involved primarily the right colon in most instances [1-5]. Only a few cases of extensive non-occlusive colonic necrosis have been reported [6-10] (Table 1). In all cases this entity has been attributed to decreased colonic perfusion but other factors could also have been involved, such as inadequate collateral circulation and increased plasma viscosity [8].

**Table 1:** Characteristics of reported cases of non-occlusive extensive colonic necrosis

**Author**	**Patient No**	**Age**	**Sex**	**Associated condition**
Renton (1967)	1	18	M	Trauma/haemorrhagic shock
Wilson (1980)	2	20	M	Trauma/haemorrhagic shock
Welch (1986)	3	50	F	Cardiac Failure/hypotension
	4	77	F	Hypotension/multiple myeloma
Levandoski (1987)	5	12	F	Trauma/haemorrhagic shock
	6	19	M	Trauma/haemorrhagic shock
	7	32	F	Tricyclic overdose/hypotension
Stockman (2006)	8	38	F	Cardiac arrest/CPR
Katsoulis (2012)	9	83	F	Cardiac arrest/CPR

In the case presented here, the most plausible explanation of extensive colonic necrosis was cardiac arrest and a period of low cardiac output despite successful CPR. This is the second report in the literature of such a combination of events. In the previous report, however, the authors speculated that the complication might have been associated with the administration of vasopressin during CPR, leading to an exaggerated visceral vasoconstrictive response [10]. Although vasopressin was not used in the present case, non-occlusive necrosis of the colon still occurred. As mentioned above, in low flow states the result of selective vasoconstriction of the mesenteric arterioles may be variable and unpredictable and non-occlusive ischaemia of the colon is one of the possible complications.

Although angiography is the gold standard imaging method for the diagnosis of acute large bowel ischaemia, MDCT with increased spatial resolution and multiplanar reformatted images has become the imaging examination of choice for the evaluation of this condition [19]. The administration of contrast intravenously allows the rapid imaging of arterial and venous phases of the mesenteric circulation. MDCT findings such as abnormalities in the bowel wall and mesentery and intraluminal haemorrhage may help in the identification of the location and the severity of acute large bowel ischaemia. Prominent bowel wall thickness, hyperdensity due to mucosal hyperaemia, inhomogeneous enhancement and intraluminal haemorrhage are findings suggesting alterations in arterial circulation [20]. Active extravasation of contrast material is defined as a hyperdense focal area (> 90 HU) within the bowel lumen in arterial phase CT images [11,21]. In alteration from impaired venous drainage, submucosal hypodensity due to oedema, pericolic streakiness and peritoneal fluid are demonstrated [20]. Intramural gas, free peritoneal air and absence of bowel wall enhancement are findings of the late stage of the disease and represent irreversible infarction and necrosis [20]. Aschoff *et al.* reported MDCT sensitivity of 93% and specificity of 100% for diagnosing mesenteric ischaemia [22].

In patients with acute abdomen and evidence of intestinal ischaemia an emergency laparotomy is warranted. The extent of bowel resection depends on the length of the necrotic bowel. Most of these patients are critically ill and anastomosis of the stumps is contraindicated particularly in cases of non-occlusive necrosis. Rapid surgery and return to the ICU are of foremost importance. In all the reported cases of extensive colonic necrosis, including the case presented here, a subtotal colectomy with end ileostomy was performed [6-10] (Table 1).

## Conclusion

Extensive ischaemia and even necrosis of the colon can occur following a period of low perfusion due to cardiogenic or hypovolaemic shock.

## Competing interests

The authors declare that they have no competing interests.

## Authors’ contributions

IEK, who was the attending surgeon, designed the study and drafted the manuscript. AB helped to draft the manuscript. MS and IG performed the literature search using the PubMEd database. AS critically revised the manuscript. MKD coordinated the study. All authors read and approved the final version.
